# Bat E-Commerce: Insights Into the Extent and Potential Implications of This Dark Trade

**DOI:** 10.3389/fvets.2021.651304

**Published:** 2021-06-10

**Authors:** Anne-Lise Chaber, Kyle N. Amstrong, Sigit Wiantoro, Vanessa Xerri, Charles Caraguel, Wayne S. J. Boardman, Torben D. Nielsen

**Affiliations:** ^1^School of Animal and Veterinary Sciences, University of Adelaide, Roseworthy, SA, Australia; ^2^School of Biological Sciences, University of Adelaide, Adelaide, SA, Australia; ^3^South Australian Museum, Adelaide, SA, Australia; ^4^Museum Zoologicum Bogoriense, Research Center for Biology, Indonesian Institute of Sciences, Cibinong, Indonesia

**Keywords:** bat taxidermy trade, bat online trade, zoonosis, conservation, biosecurity, IUCN, CITES, wildlife souvenirs

## Abstract

Little is known about the global bat souvenir trade despite previous research efforts into bat harvest for bushmeat. We screened eBay listings of bats in Australia, Canada, Italy, Switzerland, United Kingdom and USA to assess the nature and extent of the online offers. A total of 237 listings were retrieved in between the 11th and 25th of May 2020 with a median price per item of US$38.50 (range: US$8.50–2,500.00). Items on offer were mostly taxidermy (61.2%) or skull (21.1%) specimens. Overall, 32 different species of bat were advertised, most of which (*n* = 28) are listed as “Least Concern” on the International Union for Conservation of Nature (IUCN) Red List. One species (*Nycteris javanica*) is classified as “Vulnerable” and one (*Eidolon helvum*) as “Near Threatened.” *Pteropus* spp. specimens were the most expensive specimens on offer and the conservations status of these species may range from “Critically Endangered” to “Data Deficient” by IUCN and the entire genus is listed in the Appendix II by the Convention on the International Trade in Endangered Species of Wild Fauna and Flora (CITES). However, the exact species concerned, and their respective conservation status, could not be confirmed based on the listings' photos. The sourcing of bat was restricted to mostly South-East Asian countries (a third of items sourced from Indonesia) and to two African countries. Our survey revealed that the online offer of bat products is diverse, abundant, and facilitated by worldwide sellers although most offered bats species are from South-East Asia. With a few exceptions, the species on offer were of little present conservation concern, however, many unknowns remain on the potential animal welfare, biosecurity, legal implications, and most importantly public health risks associated with this dark trade.

## Introduction

Bats (order: Chiroptera) fill many ecological trophic levels and provide essential services to ecosystems in the form of pollination, seed dispersal, and insect control (1). Their presence can act as indicators of ecosystem health (2–4). With ongoing anthropogenic changes such as harvesting for bushmeat, increased agricultural expansion and deforestation the conservation of these species is increasingly compromised.

The International Union for Conservation of Nature (IUCN) Red List of Threatened Species (2020) lists 23 bat species as “critically endangered,” 60 as “endangered” and 109 as “vulnerable,” suggesting that close to 200 bat species are currently threatened with extinction. The Convention on the International Trade in Endangered Species of Wild Fauna and Flora (5) is an international agreement between governments to ensure that international trade in specimens of wild animals and plants does not threaten their existence. The species covered by CITES are listed in three appendices according to the degree of protection they need. CITES lists 11 Pteropodid species as Appendix I (no trade allowed) and two bat genera (other *Acerodon* spp., and *Pteropus* spp. not included Appendix I) in Appendix II (controlled trade) and another bat species in Appendix III (protected in at least one country).

Many bats are harvested to create collectables and souvenirs in the form of taxidermy, preserved specimens or treated skeletons. The offer of these products is suspected to extend from the streets in South East Asia into the online trade, taking advantage of e-commerce platforms such as eBay, Amazon or Etsy (6). However, the extent, and the implications, of the online trade for bat products has not been evaluated so far. It is reasonable to consider that the souvenir trade of bats may act as a conservation as well as a potential biosecurity threat.

Bats are known reservoirs of emerging and highly pathogenic viruses, some with pandemic potential (7, 8). More than 60 viruses have been detected in bat tissues and many are transmissible to humans, including Nipah-, Lyssa-, Hendra-, Ebola-, and Corona- viruses (9, 10). The wildlife trade may facilitate the exposure of viruses of zoonotic potential which can have catastrophic socioeconomic consequences, as demonstrated by the 2003 SARS outbreak, the Ebola epidemic in 2013–2016 and the current global pandemic of COVID-19.

We surveyed the offerings for bat products listed on the eBay platform in May 2020 from six different countries spread across three continents. From the sampled offers, we assessed (i) the taxonomic accuracy and diversity of the listed bats, (ii) the diversity of the items (e.g., taxidermy, dried specimens, and skeletons), and (iii) the diversity and geographical distribution of the countries of origin of the listed bats as well as the sellers. According to our findings, we have elaborated a preliminary structure of the online bat trade and discussed its potential implications on conservation and public health.

## Methods

### Online Search for Bat Products

Here, we define a “specimen” as an individual bat or part for sale, an “item” as the smallest saleable unit which may include one or several bat specimens, and a “listing” as an online entry created by a seller that describes the item/s for sale, its costs and any relevant or advertising information. The eBay homepages of Australia, Canada, Italy, Switzerland, United Kingdom and USA were interrogated for listings of bat products using the search terms “bat” and “souvenir” or “taxidermy” between the 11th and 25th of May 2020. Only listed items with whole or parts of bats (i.e., taxidermy specimens, skeletons, or skulls) and available for purchase at the time of the search were retrieved. The location and name of the seller, the species and origin of the bat on offer, the available and sold item counts, the shipping distribution and price (USD) were recorded for analysis.

### Items Speciation, Conservation Status, and CITES Listing

The pictures associated with each listed bat were examined for secondary speciation by two of the authors with extensive experience in the identification of bats from the Indo-Australasian region (KA, SW). These authors used standard external identification keys including skull and wing shapes and pelage colors. References to physical specimens at the Museum Zoologicum Bogoriense as well as to comprehensive field guides (11–13) were made where necessary. The confirmed bat species were then matched with their corresponding IUCN (14) conservation status and their CITES (5) listing. Information on threats were compiled from the current individual IUCN Red List accounts for each species listed.

### Data Management and Analyses

Data management and descriptive statistics were performed in MS Excel and IBM SPSS statistics v27. Counts and frequencies of bat products available and sold for each species and each category (i.e., skull, taxidermy, and skeleton) were summarized. Minimum, maximum and total value were converted into USD for comparison. Maps of countries distribution were generated from the retrieved list of countries using STATA v.15.1 (StataCorp Ltd., Texas, USA).

## Results

### Diversity of Online Offerings

**Figure 1:**
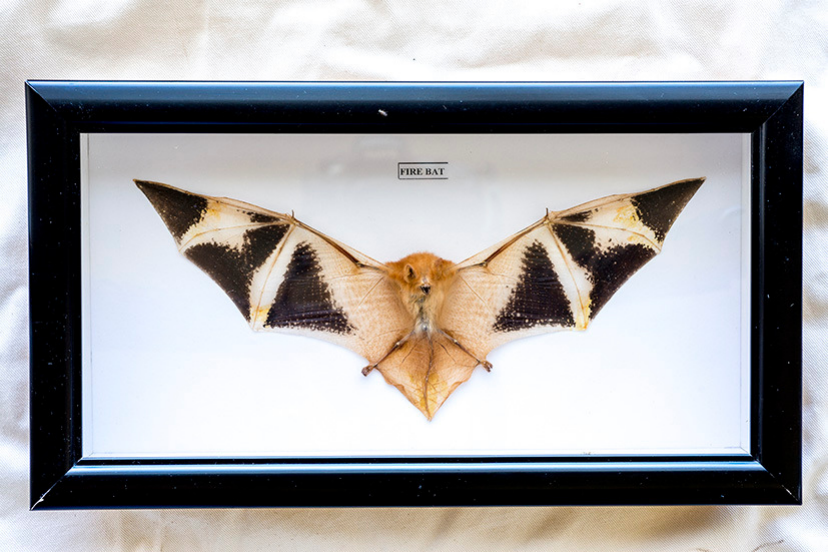
Picture of a “Fire bat” preserved in a wooden box sold on Ebay.

A total of 237 listings of bat products offered by 25 sellers were retrieved between the 11th and 25th of May 2020 from eBay websites from six countries across North America, Europe, and Australia. The median number of listings per seller was 9.5 (range: 1–91). Of the 171 listings where the count of items was described, a median of four items per listing was on offer (range: 1–46). Bat items were sold either as individual specimens or as collections of up to 50 specimens. The most common item description was taxidermy (60.2%, see example in Figure 1) followed by skull (21.1%) and skeleton (8.0%, see example in Table 1). A total of 4,467 bat specimens were on offer−1,873 taxidermy, 1,829 skulls, 516 skeletons, 239 dried bats, and 10 entomology frames. Taxidermy specimens made up 41.9% (1,873/4,467) of the total available items yet accounted for 72% of the total value of specimens available. The median price of an item was US $38.50 and ranged from US $8.50 (dried whole specimen of *Miniopterus medius*) to US $2,500.00 (whole taxidermy specimen of flying fox of the genus *Pteropus*). The next most expensive item was a whole taxidermy specimen of the advertised species *Pipistrellus javanicus* (US$1,950.00).

**Table 1:** Diversity of item's description across bat listings retrieved from eBay Australia, Canada, Italy, Switzerland, UK, USA between 11th and 25th of May 2020.

**Item description**	**Listing count (%)**	**Specimen count (%)**
Taxidermy	145 (61.2)	1,873 (41.9)
Skull	50 (21.1)	1,829 (40.9)
Skeleton	19 (8.0)	516 (11.6)
Dried	13 (5.5)	239 (5.4)
Entomology frame	10 (4.2)	10 (0.2)
Total	237 (100)	4,467 (100)

**Figure 2:**
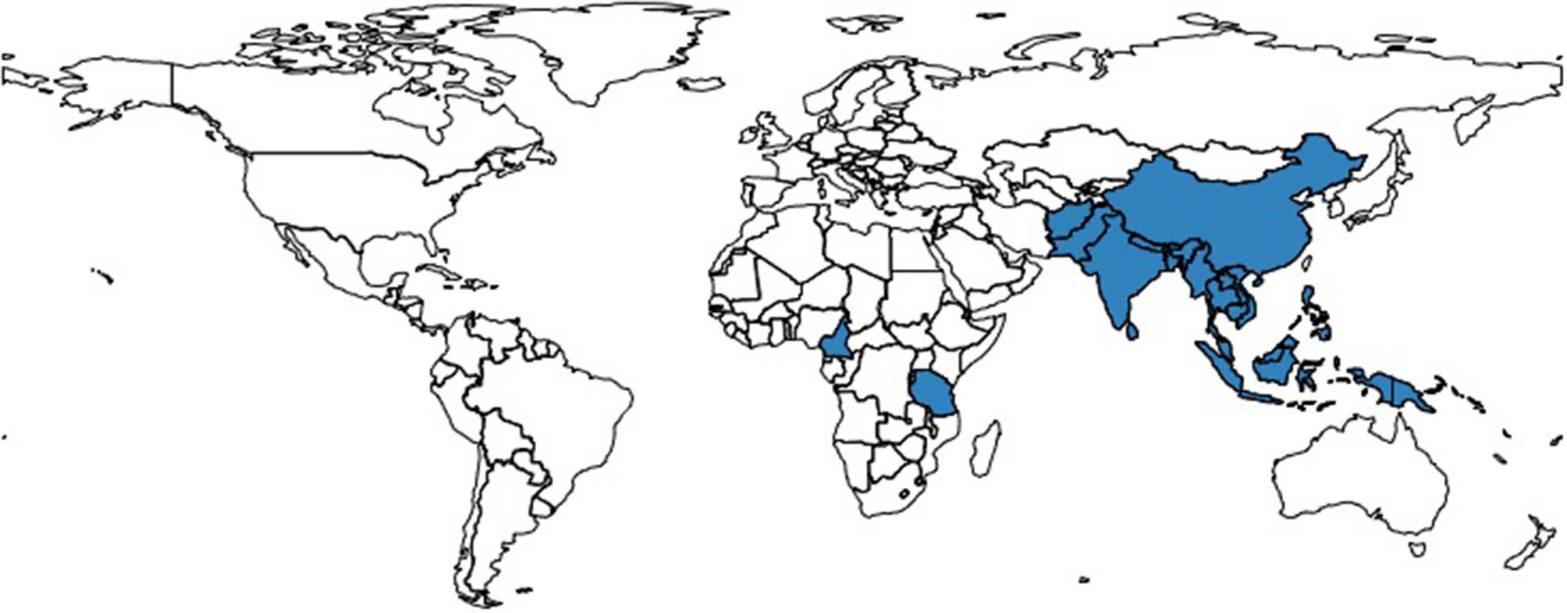
Map highlighting the advertised countries of origin of bats listed on eBay Australia, Canada, Italy, Switzerland, UK, USA between 11th and 25th of May 2020.

**Figure 3:**
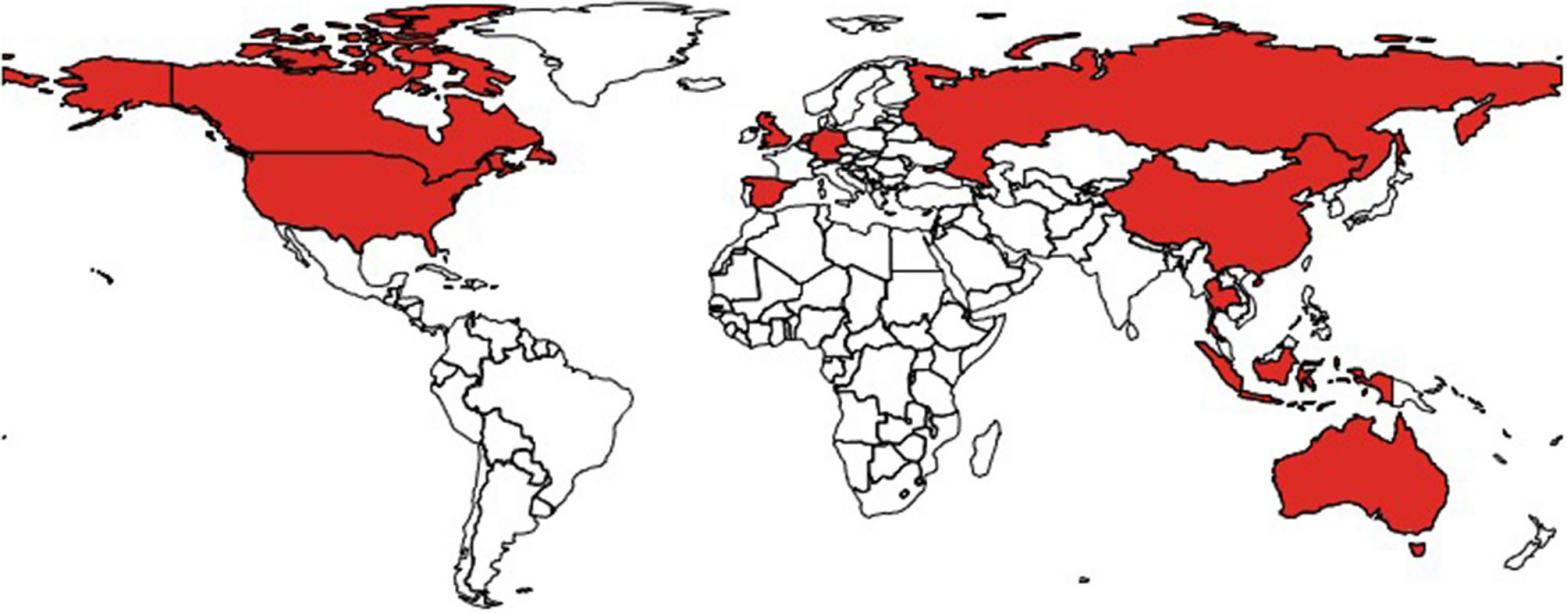
Map highlighting the advertised countries of origin of sellers offering bat items on eBay Australia, Canada, Italy, Switzerland, UK, USA between 11th and 25th of May 2020.

According to the seller's description, bats were sourced from South-East Asia (22 countries) and from Africa (two countries) (Figure 2). Approximately a third (91/247) of the listed bats were sourced in Indonesia. However, sellers' locations spanned across 11 countries in four separate continents—Australia, Canada, China, Germany, Indonesia, Netherlands, Russia, Spain, Thailand, UK, and USA (Figure 3 and Table 2). Shipping was claimed to be available worldwide in all listings but one.

**Table 2:** Geographical distribution of 24 sellers and 237 bat products retrieved from listings on eBay Australia, Canada, Italy, Switzerland, UK, USA between 11th and 25th of May 2020.

**Origin**	**Europe**	**North America**	**Asia**	**Oceania**	**Total**
Sellers (%)	10 (40.0)	8 (32.0)	5 (20.0)	2 (8.0)	25 (100)
Listing (%)	55 (23.1)	82 (34.6)	96 (40.5)	4 (1.7)	237 (100)
Countries (no of sellers, no of listings)	UK (6, 28) Spain (1, 19) Russia (1, 5) Netherlands (1, 2) Germany (1, 1)	USA (7, 57) Canada (1, 25)	Indonesia (1, 91) Thailand (3, 4) China (1, 1)	Australia (2, 4)	11 countries (25, 237)

### Species Confirmation and Conservation Status

Based on 215 listing where the seller' description include species name, a total 32 different bat species from 21 genera and 9 families were advertised (Table 3). The species *Rousettus leschenaultii* and *Hipposideros diadema* were the most frequently listed (22 listings each) followed by *Kerivoula picta* and *Eonycteris spelaea* (18 listings each), and *Cynopterus minutus* (17 listings). Of the top-5 listed species all were described as originating from Indonesia. Of the listings with examinable pictures (*n* = 204), only 33.8% (69/204) had advertised species that could be confirmed by experts and 68.6% (140/204) had the correct genus advertised. The advertised taxa of 33 listings could not be identified at all.

**Table 3:** List of bat species advertised from listings retrieved from eBay Australia, Canada, Italy, Switzerland, UK, USA between 11th and 25th of May 2020.

**Advertised species in listing**	***n*^a^**	**Confirmed taxa**	**IUCN category^b^**	**CITES listing**	**Roost type**	**Defo**	**Hunt**	**Dist**	**Mine**	**Chem**	**Fire**	**Pers**
**Pteropodidae**
*Cynopterus brachyotis*	7	Genus	LC	–	Veg	X						
*Cynopterus minutus*	17	Genus	LC	**–**	Veg							
*Cynopterus sphinx*	12	Genus	LC	**–**	Veg	X	X					
*Eidolon helvum*^c^	6	Not confirmed	NT	**–**	Veg	X	X					
*Eonycteris spelaea*	18	Species	LC	**–**	Cave	X	X					
*Hypsignathus monstrosus*^c^	7	Species	LC	**–**	Veg	X	X					
*Macroglossus minimus*	5	Species	LC	**–**	Veg							
*Micropteropus pusillus*^c^	1	Not confirmed	LC	**–**	Veg	X	X					
*Nanonycteris veldkampii*^c^	1	Not confirmed	LC	**–**	Veg							
*Pteropus* sp.	2	Genus	CE to DD	Appendix II	Veg							
*Rousettus leschenaultii*	22	Genus	LC	–	Cave		X	X				X
**Emballonuridae**
*Taphozous melanopogon*	1	Genus	LC	–	Cave	X	X	X	X			
**Megadermatidae**
*Megaderma spasma*	2	Species	LC	–	Cave	X	X	X	X			
**Nycteridae**
*Nycteris javanica*	4	Species	VU	–	Cave, veg	X						
**Hipposideridae**
*Hipposideros bicolor*	3	Species	LC	–	Cave	X		X				
*Hipposideros diadema*	22	Species	LC	–	Cave	X	X	X				
*Hipposideros larvatus*	6	Species	LC	–	Cave		X		X			
*Hipposideros madurae*	2	Genus	LC	–	Cave	X		X	X			
**Rhinolophidae**
*Rhinolophus acuminatus*	1	Genus	LC	–	Cave			X				
*Rhinolophus lepidus*	5	Genus	LC	–	Cave							
*Rhinolophus luctus*	2	Genus	LC	–	Cave	X	X					
**Vespertilionidae**
*Kerivoula pellucida*	2	Species	LC	–	Veg	X			X		X	
*Kerivoula picta*	18	Species	LC	–	Veg		X	X		X		
*Pipistrellus javanicus*	7	Genus	LC	–	Veg	X		X				
*Pipistrellus kuhlii*	6	Genus	LC	–	Veg					X		
*Pipistrellus imbricatus*	6	Genus	LC	–	Veg							
*Scotophilus kuhlii*	10	Species	LC	–	Veg		X					
*Tylonycteris pachypus*	10	Genus	LC	–	Veg	X		X			X	
*Tylonycteris robustula*	1	Genus	LC	–	Veg	X		X			X	
**Miniopteridae**
*Miniopterus medius*	9	Genus	LC	–	Cave	X		X	X			
*Miniopterus shortridgei*	1	Genus	DD	–	Cave							
**Molossidae**
*Otomops formosus*	5	Genus	DD	–	Veg							

*International Union for Conservation of Nature (IUCN) Red List of Threatened Species (2020) categories; CE, Critically Endangered; VU, Vulnerable; NT, Near Threatened; LC, Least Concern; DD, Data Deficient. Roost type; veg, vegetation. Threats to species; Defo, deforestation following logging and agriculture; Hunt, hunting for food and medicines; Dist, disturbance at roosts for a variety of reasons; Mine, includes mining and quarrying for road base; Chem, chemicals used in agriculture; Fire, fire associated with practices such as agriculture; Pers, persecution*.

a *Number of listings for each taxon. Note that a given listing may include more than one bat species*.

b *These classifications are based on the advertised species*.

c *Originating from Africa*.

Of the 32 advertised species, 27 are classified as “Least Concern” and one species (*Nycteris javanica*) is classified as “Vulnerable” by the IUCN (Table 3). One of the advertised species (*Eidolon helvum*) could not be confirmed by the experts but is classified as “Near Threatened” by IUCN. Although *Pteropus* spp. may range from “Critically Endangered” to “Data Deficient” by IUCN, the entire genus is listed in the CITES Appendix II.

## Discussion

The results presented here identified over 4,200 bat specimens on e-commerce sites from 237 listings over a 15-day period only. Bat specimens, especially taxidermy specimens, cost thousands of dollars. We discuss the risks associated with this trade to bat populations, the risks to bat health and to public health.

### Risks to Bat Populations

The online trade in bat souvenirs has the potential to add significantly to the already numerous causes of pressure on wild bat populations. Most of the species documented in this study were sourced from South East Asia, in particular Indonesia and Malaysia. The harvesting of bats for trade has been described as unsustainable and the cause of population declines (1, 15, 16). The review of the IUCN Red List profiles of all Asian species identified deforestation (47% of the 28 Asian species identified) as the most common threat, disturbance at roost sites (38%) and hunting for food and/or medicine (31%) were also commonly identified. For some species such as *Kerivoula picta*, the large demand for dried specimens and skulls as tourist souvenirs in local and foreign shops, as well as online (1), is well-known (17). The killing of bats for use as curios and souvenirs is generally undertaken at roost sites, and this represents just one of several types of disturbance at roosts, which has not been quantified. Caves are also visited for the extraction of guano, the collection of edible nests of swallows, tourism, and for the hunting of bats for food and medicine, and mining for limestone is a growing threat (18–23).

Bats are most vulnerable in their roosts because they congregate in relatively confined spaces for around half of their circadian cycle, and roosts are used as breeding sites (24). For those species offered for sale (Table 3), around half use caves and rocky overhangs as diurnal roosts, and half find refuge in tree hollows, amongst foliage, or in the hollow bases of trees. Some species use both caves and trees for roosting. Conservation assessments consider the vulnerability of species that congregate (25) and this behavior is associated with a higher risk of population decline. Bats in the families Emballonuridae, Hipposideridae, Rhinolophidae, and Miniopteridae, as well as selected medium-sized species in the Pteropodidae (species of *Eonycteris* and *Rousettus*) generally roost in caves and overhangs. Such species with large colony sizes have concentrations of individuals representing significant proportions of a population in relatively few areas, which are then relatively vulnerable to regular or catastrophic events that result from human intrusion, hunting or collecting activity. Flying-fox camps are similarly noticeable and accessible. Bat species that form smaller colonies, or that roost amongst foliage, are still vulnerable to exploitation by individuals skilled in finding them—a case in point are the colorful species of *Kerivoula* that roost within human reach in broad-leafed plants (17). Vespertilionids sometimes roost in buildings, and *Tylonycteris* roosts in bamboo, where capture is relatively straightforward.

According to the online sellers, most bats originated from Asia and especially Indonesia, where the over-exploitation of wildlife for human consumption is severe (18, 19, 26–30). This has been well-documented in Sulawesi where hunting rather than deforestation was identified as the primary cause of wildlife declines on this island. Throughout Indonesia, bats can be seen for sale in local markets. In North Sulawesi, the demand for bushmeat is so high that species such as flying foxes are imported from other provinces further south [500 metric tons per annum; (16)]. This volume is orders of magnitude greater than that supporting the international online trade in bats, but both practices continue despite the establishment of a Wildlife Crimes Unit (6), and the promotion of specific assessment efforts for threat categorization by CITES. There are simply too few resources allocated to protect wildlife against over-exploitation and flying foxes are not considered as threatened by the Indonesian government. Indonesian law allows the hunting and trading of unprotected animals such as bats, but there is a legal permit (Law Number 5, year 1990; Government Act Number 8, year 1999) that is seldom enforced at a local level. Legally mandated quotas for inter-provincial trade of unprotected animals have also not been implemented (16). Within the context of hunting for bushmeat it appears that precipitating direct action against the relatively few individuals involved in the online trade of bat souvenirs will be challenging, unless other risks such as biosecurity can be usefully applied.

### Infectious Disease Risks

The methods by which the listed bats were sourced, collected, and killed for the purpose of taxidermy are unknown. Many of these listings did not specify the taxidermy method used, rather most listings advertised the specimen as “dried.” No further information regarding the processing method was provided. Indeed, knowledge of the procedures, including biosecurity protocols for the preparation of bat taxidermy specimens is limited and as a consequence the infectious disease risks during processing, exportation and importation are unknown. To more fully understand the risks, there is a requirement to understand which species act as reservoirs of infectious disease agents of zoonotic potential and the ability for the infectious disease agents to spillover from handled live bats and for them to survive and remain infectious in the tissues of the harvested animals and in contact materials.

#### Risks to Bat Health

The route and likelihood of transmission of pathogens to bats from the online bat trade is not obvious and specimens and curios will presumably remain in people's collections. One of the greatest biosecurity threats identified recently to bats is the pathogenic fungus *Pseudogymnoascus destructans* that causes White nose syndrome and has led to significant losses of bats in North America (31).The greatest risk of entry into countries that are free of this pathogen such as Australia is from fomites on unclean caving equipment brought in from North America, Europe or north-eastern China. At least seven Australian species of bat have been identified as susceptible to exposure (32). While the likelihood of the introduction of a foreign pathogen to native Australian bats from the online trade in bat souvenirs might be low, the consequences could be catastrophic, and place additional pressure on critically endangered species that are already declining from a range of threats (33).

#### Risks to Public Health

Individuals having contact with wildlife bear the greatest risk of contracting novel infectious agents, since transmission often occurs via bites, scratches, and exposure to body fluids, tissues and excrement (9). Therefore, trade that brings wildlife into close proximity to humans enhances the risk of pathogen transmission. It is unknown, but we suggest unlikely, that bat harvesters are using effective personal protective equipment (PPE) that protects them against bites, scratches or secretions. In a study conducted by Kamins et al. (9), that investigated Ghanaians' understanding of bat-borne diseases, 86% of participants who hunted bats did not believe handling them posed any sort of risk. If similar beliefs are held by those harvesting bats for taxidermy, it is likely they are at risk of a zoonotic infection. Observations of bush meat preparation in Laos, revealed only one bat vendor washed their hands, only four of the seven markets had access to running water, and most markets stalls showed evidence of blood or entrails (34). Individuals preparing bats for taxidermy might follow similar poor hygiene standards to those in Laos or Ghana and could be exposed to zoonotic risks. Most of the specimens listed online are of South East Asian origin and this region is considered a hotspot for emerging infectious diseases of zoonotic potential, in part because it contains 30% of the known global bat species (35).

There are several species of zoonotic viruses that may constitute a risk to public health along the harvesting—processing—seller—buyer continuum including Lyssaviruses, Henipaviruses, Ebolaviruses, and Coronaviruses (Table 4). Transmission of lyssaviruses commonly occurs following contact with infected saliva or neural tissue. As the virus can survive in saliva for 24 h and, is likely to persist within the neural tissue, harvesters and taxidermists are at possible risk of zoonosis if safety precautions are not taken (58). Environmental contamination by lyssavirus infected bats is considered negligible, since the classical rabies virus is fragile and does not survive for long outside the host. Henipaviruses, including Hendra virus and Nipah virus, are a group of highly pathogenic zoonotic viruses. Hendra virus has caused disease in Australia in horses and humans and Nipah virus has caused disease in pigs and humans in Malaysia and directly in humans in Bangladesh and India. Flying foxes are the reservoir hosts (59) and viruses can be transmitted to humans directly or indirectly via contact with saliva, urine or other bodily fluids. One specimen from each of these taxa is available for purchase online, excluding *E. helvum*, where there are nine specimens available. Fogarty et al. (39) investigated the persistence of Henipaviruses under various environmental conditions in a laboratory setting (Table 4). The risks of transmission to a new host requires close contact with an infected bat or exposure to contaminated material shortly after excretion. Under optimal conditions it is possible for the virus to persist for several days and fomite transmission may be possible under these circumstances. Ebola viruses, members of the Filoviridae family, are highly pathogenic and are associated with infection in bats in Africa (43, 60) included the Hammer-headed bat (*Hypsignathus monstrous*) which is listed seven times as available for purchase on eBay [(61); see Table 4]. Human transmission is possible via direct contact between mucous membranes, open wounds and excreta, bites and consumption of contaminated fruit and other objects (43). Persistence of the virus in dried blood for 5 days and in feces for up to 21 days suggests harvesters, taxidermists, sellers and buyers might be at risk. Coronaviruses (CoV) are capable of causing severe enteric and respiratory diseases in domestic animals and humans and include SARS-CoV-1, MERS-CoV, and SARS-CoV-2 (62, 63). Bats including *Rhinolophus* spp. are thought to be the reservoirs of the SARS coronaviruses (63, 64). This genus was identified in over 380 listings. Reports of coronavirus survival in the environment is limited (see Table 4), however viability can be prolonged in optimal conditions and therefore we consider that coronavirus could pose a risk to harvesters, taxidermists, sellers, and buyers.

**Table 4:** Examples of bat associated viruses of public health concern, their hosts, excretion pathways, and factors affecting the virus's survival.

**Virus family**	**Examples of bat associated virus of known public health concern**	**Associated bat reservoir host taxa**	**Excretion and transmission pathways of public health concern**	**Factors affecting survival of virus outside the host**
Paramyxoviruses	Hendra virus (HeV)	*Pteropus alecto*^a^ *Pteropus conspicallatus*^a^ *Eidolon helvum*^b^	*Pteropus alecto* In order of decreasing risk—urine, blood, feces, nasal discharge, saliva^a^ Urine-oronasal^c^ saliva to intermediary species	Stable in urine for 4 days at 22°C at pH = 7^d^ Inactivated <1 day at 37°C Increasingly inactivated by desiccation plus temperatures < or >22°C and pH < or > pH = 7^d^ 19 h half-life in urine pH = 7^d^ HeV and NiV broad tolerance to pH changes^d^
	Nipah virus (NiV)	*Pteropus vampyrus*^e^ *Pteropus hypomelanus*	Urine, saliva to intermediary species or, aerosol, saliva (food) direct^f^	Stable in blood for 3 days at 20 or 30°C^g^ 18 h half-life in urine pH = 7^d^ HeV and NiV broad tolerance to pH changes^d^
Filoviruses	Ebola Zaire virus	*Hypsignathus monstrous* *Epomops franqueti* *Myonycteris torquata* *Micropteropus pusillus* *Mops condylurus* *Rousettus aegyptiacus*^h^	Saliva/Aerosol to wounds, mucous membranes, via fruit^i^^,^ ^j^	Stable in dried blood for 4–5 days^k^ Persistence in feces for 21 days post-infection^l^
	Marburg virus	*Rousettus aegyptiacusHypsignathus monstrous*^i^		Stable for 5 days on a surface^m^
Coronaviruses	SARS-CoV-1	*Rhinolophus* spp.^n^ *Scotophilus* spp.^o^	Droplets, fomites, fecal-oral^p^	Stable for 5 days at 22–25°C^q^
	MERS-CoV	*Taphozus perforatus* *Rhinopoma hardwickii* *Pipistrellus kuhlii*^r^	Droplets, fomites^p^	Stable for 2 days at 20°C and 40% RH^s^ Inactivated at temperatures >30°C and high relative humidity^s^
	SARS-CoV-2	*Rhinolophus* spp.^t^	Droplets, fomites, fecal-oral^p^	Stable 5 days on metal, 5 days on paper^u^
	HCoV-229E & HCoV-NL63	*Hipposiderid* spp.^l^ *Perimyotis subflavus*^v^	Droplets, fomites^p^	
Rhabdoviruses	Rabies virus^u^	American bats^w^	Bite/scratch associated with saliva	Stable in saliva for 24 h at 0–4°C Inactivated by heat and direct sun Bats dead >4 h not infectious
	European bat lyssa virus 1 & 2^u^	European bats^w^	Bite/scratch associated with saliva	Likely similar to rabies virus
	Lagos bat lyssavirus^u^	African bats^w^	Bite/scratch associated with saliva	Likely similar to rabies virus
	Duvenhage bat lyssavirus^u^	African bats^w^	Bite/scratch associated with saliva	Likely similar to rabies virus
	Australian bat lyssavirus^u^	*Pteropus* spp.*Saccolaimus* spp.	Bite/scratch associated with saliva	Likely similar to rabies virus

a *Edson et al. (36)*.

b *Hayman et al. (37)*.

c *Field (38)*.

d *Fogarty et al. (39)*.

e *Moratelli and Calisher (7)*.

f *Sayed et al. (40)*.

g *Smither et al. (41)*.

h *Pourrut et al. (42)*.

i *Markotter et al. (43)*.

j *Piercy et al. (44)*.

k *Sinclair et al. (45)*.

l *Swanepoel et al. (46)*.

m *Belanov et al. (47)*.

n *Banerjee et al. (48)*.

o *Chen et al. (49)*.

p *Ye et al. (50)*.

q *Chan et al. (51)*.

r *Anthony et al. (52)*.

s *van Doremalen et al. (53)*.

t *Zheng (54)*.

u *Wiktorczyk-Kapischke et al. (55)*.

v *Huynh et al. (56)*.

w *Constantine (57)*.

Transmission of bacterial pathogens from bats to humans via direct or indirect contact is also possible and may affect bat harvesters and taxidermists. The zoonotic potential of bacterial pathogens from bats to humans is less well-studied (65, 66) but bacterial diseases, such as Bartonellosis and Leptospirosis and diseases associated with enteric bacteria are common human infections that have also been detected in bats. As an example, Leptospirosis is reported to colonize the kidneys of infected bats and can be excreted in urine. The estimated prevalence within bat populations varies between 2 and 35% (65). Similarly, studies have reported that enteric bacteria, *Salmonella* spp. and *Escherichia coli* isolated from infected bats share similar characteristics with those from infected humans suggesting the infection was from bat origins (65, 67).

Without understanding the methodology of harvesting and the specific processing and treatment conditions the bats undergo before being listed for sale online, it is reasonable to suggest that on-line bat trade could pose a human health risk. The potential for viral recombination events needs to be considered as we have no understanding if harvesters keep species alive before processing. At this stage the risks cannot be qualitatively determined without undertaking a detailed risk assessment process which is outside the brief of this paper. Nonetheless, this preliminary study shows that specimens can move from the point of harvesting, to processors, to sellers and then onto buyers and these actions could pose a potential health risk.

##### Legislation, Biosecurity, and Permits

Import and export legislations differ in each country. Australia has a relatively strict biosecurity control infrastructure and legal instruments and will be used as an example for the purpose of this discussion. Certain commodities are only allowed legal entry into Australia upon the importer being granted an import permit from the Department of Agriculture, Water and the Environment (DAWE) but taxidermy specimens imported for personal use may be imported without a permit if the specimen is fully tanned, free of adhering fat, muscle, bone and evidence of decay. If the specimen of interest is a skull, horn or skeleton, it must be boiled. Lastly, prior to the arrival and subsequent entry into Australian territory the specimen must be clean and free of contaminants such as seed, soil, animal and plant debris, and other biosecurity risk material. In addition, the Environment Protection and Biodiversity Act 1999 (EPBC) outlines laws and regulations that assist in the protection of the environment from biosecurity risks associated with the importation of wildlife from overseas. The EPBC Act further allows Australia to meet the international agreement outlined by the Convention of International Trade in Endangered Species of Wild Fauna and Flora (CITES) (68). CITES was implemented to ensure the international trade in plants and wild animals does not threaten their survival. None of the import requirements were highlighted on the e-advertisements and it is difficult, without physical examination of a specimen(s) to accurately identify it and ensure that they are indeed fully tanned or simply just dried.

## Conclusion

This preliminary study identifies that over a 15-day period in May 2020, 4,467 bat specimens reflecting 32 different species sourced from 24 countries were being traded online. They included species that are considered threatened with extinction and taxa that are listed on Appendix II of CITES. The threat to conservation and biodiversity is evident and is a genuine concern due to bats' important role in ecosystems. Current legislation exists to prevent the movement and trade of threatened species as well as CITES listed animals, including bats, into and out of Australia, Canada, Europe, and the United Kingdom but enforcement is challenging due to the high volume of items traded on the internet and shipped daily. The study also highlights that as bats are known to be reservoir hosts of significant zoonotic viruses, that spillover events associated with this trade could possibly occur especially Ebolaviruses and Coronaviruses which can remain stable for some time in variable conditions. As most pathogen spillover events have wildlife trade origins, it is important we improve our understanding of this specific wildlife trade; bat E-commerce, with respect to viral transmission and how to adjust current social and cultural practices in order to mitigate the risk of zoonoses.

The data presented is most likely a vast underestimation of the true extent of the online bat trade. We suggest further studies are required to determine the sustainability of this trade, its welfare implications and the health risks posed to harvesters, taxidermists, sellers and buyers. By getting a better understanding of the extent of this commodity chain and which species are involved, it may help to identify higher risk groups for zoonotic transmission. Research should include a detailed risk analysis following accepted international guidelines, investigating the buyer's awareness, attitude and perception of bat trading to assess whether they understand the health and conservation risks, reviewing importation laws and what extension education services can be provided to reduce the trade and associated risks. To ignore this particular type of wildlife trade, Bat E-commerce, risks the emergence of spillover events into humans that could have far reaching consequences.

## Data Availability Statement

The original contributions presented in the study are included in the article/supplementary material, further inquiries can be directed to the corresponding author/s.

## Author Contributions

A-LC conceived of the original idea. VX and A-LC performed the data mining. KA and SW identified the species on sale. CC and TN analyzed the data. A-LC, VX, and KA wrote the manuscript with support from TN. WB, SW, CC, and A-LC supervised the project. All authors contributed to the article and approved the submitted version.

## Conflict of Interest

The authors declare that the research was conducted in the absence of any commercial or financial relationships that could be construed as a potential conflict of interest.
